# Cost-effectiveness of ursodeoxycholic acid in preventing new-onset symptomatic gallstone disease after Roux-en-Y gastric bypass surgery

**DOI:** 10.1093/bjs/znac273

**Published:** 2022-08-18

**Authors:** Sylke Haal, Maimoena S S Guman, L Maurits de Brauw, Ruben Schouten, Ruben N van Veen, Paul Fockens, Victor E A Gerdes, Rogier P Voermans, Marcel G W Dijkgraaf

**Affiliations:** Department of Internal Medicine, Spaarne Gasthuis, Hoofddorp, the Netherlands; Department of Gastroenterology and Hepatology, Amsterdam UMC, University of Amsterdam, Amsterdam Gastroenterology Endocrinology Metabolism, Amsterdam, the Netherlands; Department of Internal Medicine, Spaarne Gasthuis, Hoofddorp, the Netherlands; Department of Internal and Vascular Medicine, Amsterdam UMC, University of Amsterdam, Amsterdam Gastroenterology Endocrinology Metabolism, Amsterdam, the Netherlands; Department of Surgery, Spaarne Gasthuis, Hoofddorp, the Netherlands; Department of Surgery, Flevohospital, Almere, the Netherlands; Department of Surgery, OLVG, Amsterdam, the Netherlands; Department of Gastroenterology and Hepatology, Amsterdam UMC, University of Amsterdam, Amsterdam Gastroenterology Endocrinology Metabolism, Amsterdam, the Netherlands; Department of Internal Medicine, Spaarne Gasthuis, Hoofddorp, the Netherlands; Department of Internal and Vascular Medicine, Amsterdam UMC, University of Amsterdam, Amsterdam Gastroenterology Endocrinology Metabolism, Amsterdam, the Netherlands; Department of Gastroenterology and Hepatology, Amsterdam UMC, University of Amsterdam, Amsterdam Gastroenterology Endocrinology Metabolism, Amsterdam, the Netherlands; Department of Epidemiology and Data Science, Amsterdam UMC, University of Amsterdam, Amsterdam, The Netherlands; Amsterdam Public Health, Methodology, Amsterdam, the Netherlands

## Abstract

**Background:**

The aim was to evaluate the cost-effectiveness and cost–utility of ursodeoxycholic acid (UDCA) prophylaxis for the prevention of symptomatic gallstone disease after Roux-en-Y gastric bypass (RYGB) in patients without gallstones before surgery.

**Methods:**

Data from a multicentre, double-blind, randomized placebo-controlled superiority trial were used. Patients scheduled for laparoscopic RYGB or sleeve gastrectomy were randomized to receive 900 mg UDCA or placebo for 6 months. Indicated by the clinical report, prophylactic prescription of UDCA was evaluated economically against placebo from a healthcare and societal perspective for the subgroup of patients without gallstones before surgery who underwent RYGB. Volumes and costs of in-hospital care, out-of-hospital care, out-of-pocket expenses, and productivity loss were assessed. Main outcomes were the costs per patient free from symptomatic gallstone disease and the costs per quality-adjusted life-year (QALY).

**Results:**

Patients receiving UDCA prophylaxis were more likely to remain free from symptomatic gallstone disease (relative risk 1.06, 95 per cent c.i. 1.02 to 1.11; *P* = 0.002) compared with patients in the placebo group. The gain in QALYs, corrected for a baseline difference in health utility, was 0.047 (95 per cent bias-corrected and accelerated (Bca) c.i. 0.007 to 0.088) higher (*P* = 0.022). Differences in costs were –€356 (95 per cent Bca c.i. €–1573 to 761) from a healthcare perspective and –€1392 (–3807 to 917) from a societal perspective including out-of-pocket expenses and productivity loss, both statistically non-significant, in favour of UDCA prophylaxis. The probability of UDCA prophylaxis being cost-effective was at least 0.872.

**Conclusion:**

UDCA prophylaxis after RYGB in patients without gallstones before surgery was cost-effective.

## Teaser

This cost-effectiveness analysis compared ursodeoxycholic acid (UDCA) prophylaxis with placebo for the prevention of symptomatic gallstone disease after Roux-en-Y gastric bypass . UDCA prophylaxis resulted in a significantly higher proportion of patients remaining free from symptomatic gallstone disease and in a surplus of nearly 0.05 in QALYs gained from baseline. The mean cost savings of €1392 was not significant.

## Introduction

Patients with morbid obesity undergoing bariatric surgery are at risk of developing cholesterol gallstones^1^. Of patients who have bariatric surgery, up to 40 per cent develop cholesterol gallstones^2–4^ and approximately 8–15 per cent become symptomatic^5–7^. Most symptomatic patients develop biliary colic for which elective laparoscopic cholecystectomy is indicated to reduce the risk of recurrence and severe biliary disease. Less commonly, patients present directly with severe disease, such as cholecystitis, cholangitis, choledocholithiasis, or biliary pancreatitis, which requires admission to hospital and various diagnostic and therapeutic procedures. As a result of the ongoing obesity epidemic and rise in bariatric interventions, the disease and financial burden of symptomatic gallstone disease will increase further.

An opportunity to prevent gallstone formation medically during rapid weight loss is provided by the prophylactic administration of ursodeoxycholic acid (UDCA), an oral bile acid. Meta-analyses^8,9^ have shown that UDCA prophylaxis can prevent the formation of cholesterol gallstones during rapid weight loss induced by very low-calorie diets or bariatric surgery. However, the prescription of UDCA prophylaxis has been debated, as most patients with gallstones remain asymptomatic and there has been little evidence available regarding the efficacy of UDCA in preventing symptomatic gallstone disease. Recently, the UPGRADE trial^10^ showed that UDCA prophylaxis for 6 months reduced the occurrence of symptomatic gallstone disease compared with placebo in patients without gallstones before Roux-en-Y gastric bypass (RYGB). A barrier to worldwide implementation could be the costs associated with UDCA prophylaxis. Therefore, this study aimed to evaluate the cost-effectiveness and cost–utility of UDCA prophylaxis for the prevention of symptomatic gallstone disease after RYGB in patients without gallstones before operation, using data from the multicentre UPGRADE trial.

## Methods

### UPGRADE trial

This economic evaluation was performed alongside the UPGRADE trial, a multicentre, double-blind, randomized, placebo-controlled superiority trial. Details of the design, statistical analysis plan, and clinical results have been reported previously^10–12^. The institutional review board of Slotervaart Hospital and Reade (Amsterdam, the Netherlands) approved the trial protocol. In summary, patients with morbid obesity and an intact gallbladder scheduled for laparoscopic RYGB or sleeve gastrectomy were eligible for inclusion. Patients were assigned randomly to receive either 900 mg UDCA daily or placebo for 6 months in a 1 : 1 ratio. The primary endpoint was symptomatic gallstone disease within 24 months. Prophylactic prescription of UDCA was evaluated economically for the subgroup of patients without gallstones before surgery, who underwent RYGB and who had data available for primary endpoint assessment at 24 months^13^.

### Type of health economic evaluation, outcomes, perspective, and time horizon

The economic evaluation was performed as cost-effectiveness (CEA) and cost–utility (CUA) analyses from a healthcare and societal perspective with a time horizon of 2 years. The comparator for UDCA prophylaxis was placebo treatment. The CEA endpoint was the costs per patient free from symptomatic gallstone disease for 24 months. The CUA endpoint was the costs per quality-adjusted life-year (QALY). All relevant healthcare costs, out-of-pocket expenses incurred by patients, and productivity loss were assessed during a follow-up of 24 months. Considering the time horizon of 2 years after bariatric surgery, QALYs in the second year of follow-up were discounted by 1.5 per cent, and costs were discounted by 3.5 and 4 per cent. The CHEERS statement was followed for the reporting of this study^14^.

### Cost components, resources and unit costing

Healthcare costs included costs that were possibly differentially affected by prophylactic UDCA and placebo treatment, and encompassed the costs of the prescription of UDCA, postdischarge emergency department visits, repeat hospital admissions (including stay on ward, ICU, coronary care unit, nursing day care), diagnostic (such as imaging, laboratory tests, and preoperative gallbladder ultrasonography) and therapeutic (endoscopic retrograde cholangiopancreatography, cholecystectomy) procedures, outpatient hospital consultations, out-of-hospital consultations (by general practitioner, company physician, and psychologist), formal home care (housekeeping, personal care, and nursing), and daily use of wound or stoma materials during 24 months of follow-up. Out-of-pocket expenses assessed comprised over-the-counter medication, non-reimbursable dietaries, and private home help. Productivity loss among patients with a paid job owing to sick leave (absenteeism) or lowered efficiency while at work (presenteeism) was also assessed.

Data on hospital care volume were gathered from the day after hospital discharge following index surgery until 24 months after the index surgery. Data were mostly extracted from hospital information systems and to a lesser extent from case report forms (cholecystectomies in hospitals not participating as a research location). Initially, three hospitals participated in the UPGRADE trial, but two of these went bankrupt unexpectedly and were replaced by two new centres that continued care for the study participants. Hospital care volume data were retrieved from all participating centres except for the bankrupt hospital with the smallest number of patients (73). Information on inpatient stay on wards or ICUs elsewhere, as well as emergency department visits elsewhere (in non-research locations), was retrieved from self-reported questionnaires. The iMTA Medical Consumption Questionnaire (iMCQ) was adjusted for the target population and study setting, and used to gather data on volumes of (out-of-)hospital care^15^. The iMTA Productivity Costs Questionnaire (iPCQ) was used to gather data on job status, absenteeism from work, and presenteeism while at work^15^. The questionnaires were sent to patients before surgery (baseline), and at 3, 6, 12, 18, and 24 months after operation. Items on monthly out-of-pocket expenses were added to this battery. The timing of questionnaires was based on the expected disease dynamics and research burden for patients. Where recall periods in questionnaire items were shorter than the time between successive measurements, the multiplier ‘time between measurements divided by recall period’ was used to weigh the assessments at different time points.

Unit costs of resources used and their sources are shown in *Table S1*. Unit costs were derived from the 2021 Dutch Pharmaceutical Compass, the 2015 Dutch manual for costing in healthcare research^16^, hospital ledger data, and unit costs of mutual services, based on availability and appropriateness. The unit cost of UDCA held for the full course over 182 days. All unit costs are expressed in euros for the reference year 2019 after price indexing with general consumer price index figures for the Netherlands if sources with different base years were used^17^.

Costs were calculated as the sum of the products of volumes of resources used and their respective unit costs. Preoperative ultrasonography of the gallbladder is needed to identify patients without gallstones before surgery. Asymptomatic gallstones are diagnosed in about 20 per cent of patients with an intact gallbladder scheduled for bariatric surgery. The higher unit costs of ultrasonography of the abdomen rather than target ultrasonography of the gallbladder were taken to compensate for the extra screening costs for patients who were eventually not eligible for UDCA prophylaxis. Productivity loss from absenteeism lasting longer than the friction period of 109 days during the observation years of the UPGRADE trial was not included, assuming that the absentee was fully replaced by another worker from the pool of unemployed. Working hours lost by presenteeism were assessed by multiplying the reported number of days of presenteeism by the mean baseline hours per working day and the factor (10 – *x*)/10, where *x* represents the patient-reported level of efficiency ranging from 0 (nothing accomplished) to 10 (worked as usual).

### Health effects

The health effect for the CEA was staying free from symptomatic gallstone disease for 24 months after surgery, and was assessed during follow-up in the UPGRADE trial. The EuroQol (EQ) 5D-5L™ questionnaire (EuroQol Group, Rotterdam, the Netherlands) was completed by patients before surgery (baseline), and at 3, 6, 12, 18, and 24 months after operation. EQ-5D-5L™ health status scoring profiles were converted into health utilities using the Dutch EQ-5D-5L™ tariff^18^. Patients who died were assigned a health utility score of 0 for the remainder of follow-up. Because the baseline health utility score differed significantly between the study groups, the change in QALYs per patient was calculated by multiplying the baseline health utility score by 2 as the reference situation, and by subsequently subtracting this quantity from the calculated area under the curve of health utility scores during follow-up.

### Handling of missing data

Multiple imputation by fully conditional specification using predictive mean matching was applied to handle missing health utility data during follow-up. Five multiply imputed sets were created with baseline characteristics (including baseline health utility), the assigned treatment (UDCA or placebo), the incidence of adverse events during follow-up, BMI during follow-up, adherence to therapy, the result of postoperative ultrasonography, and the occurrence of symptomatic gallstone disease and cholecystectomy as predictors. The mean of five imputed scores for any missing health utility measurement was taken as the final proxy score.

Missing volume data from the iMCQ and iPCQ, and out-of-pocket expenses were imputed using the mean scores for available data at the time of measurement in each study group. Missing data from hospital registries were imputed likewise, but only at subaggregate level (number and costs of inpatient hospital days, outpatient hospital consultations, emergency department visits, and diagnostic and therapeutic procedures) covering the full follow-up interval.

### Statistical analysis

Categorical baseline characteristics were compared using the χ^2^ test. The independent-samples *t* test was used for normally distributed continuous variables. Costs and health effects per study group, as well the differences in costs and health effects between UDCA prophylaxis and placebo groups, are reported with bias-corrected and accelerated (Bca) 95 per cent confidence intervals, and the independent samples *t* test was used to evaluate differences (SPSS^®^ version 24.0; IBM, Armonk, NY, USA). These confidence intervals were generated by non-parametric bootstrapping, drawing 5000 samples of the same sizes as the original study groups and with replacement, stratified by study group^19^.

The incremental CEA and CUA were done for the extra costs per additional patient free from symptomatic gallstone disease and the extra costs per QALY gained respectively, again following the non-parametric bootstrapping procedure. Results are visualized using cost-effectiveness planes and cost-effectiveness acceptability curves for societal willingness to pay per additional patient free from symptomatic gallstone disease up to €10 000 and per additional QALY up to €20 000.

A sensitivity analysis was undertaken for two distinct UDCA treatment costs. Undiscounted results were taken as the base case, with discounted results addressed in a sensitivity analysis.

## Results

### Baseline characteristics

Between January 2017 and October 2018, 985 patients were enrolled in the UPGRADE trial; 959 patients were included in the modified intention-to-treat analysis and had data available for primary endpoint assessments at 24 months. Both the presence of asymptomatic gallstones before surgery and type of surgery were predefined points of interest. The clinical results showed that UDCA prophylaxis was of no relevance to patients with asymptomatic gallstones before surgery (186 people)^10^. A suggested benefit of UDCA prophylaxis for patients receiving a sleeve gastrectomy (78) could not be confirmed^10^. Efficacy was confirmed only in the remaining 705 patients without gallstones before surgery who underwent RYGB. The baseline characteristics of these 705 patients are reported in *Table 1*. Baseline characteristics of the UDCA group (348 patients) and placebo group (357) were comparable, except for the health utility score, which indicated that patients having UDCA prophylaxis experienced more health-related problems than those receiving placebo (mean difference −0.028, 95 per cent Bca c.i. −0.052 to –0.003; *P* = 0.027).

**Table 1:** Baseline characteristics of patients without gallstones before surgery, who underwent Roux-en-Y gastric bypass, and who had data available for primary endpoint assessment at 24 months

	UDCA group (*n* = 348)	Placebo group (*n* = 357)
**Age (years), mean(s.d.)**	44.9 (11.2)	44.1 (11.2)
**Sex**		
F	267 (76.7)	271 (75.9)
M	81 (23.3)	86 (24.1)
**Weight (kg), mean(s.d.)**	115.5 (18.9)	115.6 (17.2)
**BMI (kg/m^2^), mean(s.d.)**	39.8 (4.6)	40.0 (4.6)
**Co-morbidities**		
Hypertension	167 (48.0)	173 (48.5)
Dyslipidaemia	105 (30.2)	124 (34.7)
Type 2 diabetes	48 (13.8)	68 (19.0)
**Statin use at baseline**	58 (16.7)	69 (19.3)
**Health utility score, mean(s.d.)** *	0.833 (0.181)	0.861 (0.150)

Values are *n* (%) unless otherwise indicated. *Based on the scoring profiles of the EQ-5D-5L^™^ questionnaire. Observed health utility scores range from −0.05 (health state worse than death) to 1 (perfect health state). Mean difference −0.028 (95 per cent bias-corrected and accelerated c.i. −0.052 to −0.003; *P* = 0.027, independent-samples *t* test). UDCA, ursodeoxycholic acid.

### Differences in health

Of patients receiving UDCA prophylaxis, 336 of 348 (96.6 per cent) remained free from symptomatic gallstone disease compared with 324 of 357 (90.8 per cent) in the placebo group (relative risk 1.06, 95 per cent c.i. 1.02 to 1.11; *P* = 0.002).

Patients in the UDCA and placebo groups gained 0.120 (95 per cent Bca c.i. 0.092 to 0.147) and 0.073 (0.042 to 0.103) QALYs over 2 years respectively (compared with if they had remained stable over time at their baseline utility scores); the mean difference was 0.047 (0.007 to 0.088; *P* = 0.022), which was clinically relevant (*Table 1*).

### Differences in volumes and costs

*Table 2* shows the mean use of healthcare resources by study group. Closely reflecting the higher proportion of patients remaining free from symptomatic gallstone disease, there were fewer cholecystectomies in the UDCA group. There were also fewer diagnostic and therapeutic procedures, and fewer visits to the emergency department in the UDCA group.

**Table 2:** Mean use of resources per patient by study group during patient selection and after discharge from index admission following Roux-en-Y gastric bypass

	Mean volume per patient		
	UDCA group (*n* = 348)	Placebo group (*n* = 357)	Difference UDCA – placebo
**Hospital care**			
Patient selection and intervention			
Preoperative gallbladder ultrasonography	1	–	1
UDCA for 182 days	1	–	1
Laparoscopic cholecystectomy	0.032 (0.017, 0.046)	0.092 (0.064, 0.12)	−0.061 (−0.095, −0.027)
Diagnostic and therapeutic procedures*	58 (54, 62)	65 (60, 70)	−6.7 (−13.1, −0.4)
Outpatient specialist consultations	5.3 (4.9, 5.7)	5.4 (5, 5.9)	−0.1 (−0.7, 0.5)
Emergency department visits	0.46 (0.37, 0.56)	0.72 (0.60, 0.86)	−0.26 (−0.43, −0.09)
Duration of inpatient stay (days)†	1.32 (0.94, 1.79)	1.75 (1.28, 2.35)	−0.43 (−1.24, 0.34)
**Out-of-hospital care**			
Number of consultations	3.7 (2.9, 4.5)	3.7 (2.9, 4.7)	0 (−1.4, 1.2)
Time spent in other institutional care (days)	0.03 (0.002, 0.07)	0.17 (0.02, 0.44)	−0.15 (−0.55, 0.05)
Duration of formal home care (h)	6 (3, 11)	19 (7, 34)	−12 (−32, 2)
Duration of use of wound or stoma materials (days)	1.1 (0.5, 2)	0.9 (0.4, 1.4)	0.3 (−0.7, 1.3)
**Productivity loss (h)‡**	180 (146, 216)	208 (171, 250)	−28 (−82, 24)
Absenteeism	128 (102, 155)	162 (132, 196)	−35 (−78, 7)
Presenteeism	52.0 (39, 66)	46 (35, 59)	6 (−12, 25)

Values in parentheses are 95 per cent bias-corrected and accelerated confidence intervals. Testing for differences is based on cost data.

* Data on diagnostic and therapeutic procedures could be retrieved from hospital information systems for 299 of 348 patients in the ursodeoxycholic acid (UDCA) group and 300 of 357 in the placebo group.

†Includes stay in general ward, ICU, coronary care unit, and day nursing.

‡Some 247 patients in the UDCA group and 258 in the placebo group had paid jobs at baseline; data on absenteeism and presenteeism were missing for 49 of 1235 (4.0 per cent) and 83 of 1290 (6.4 per cent) planned follow-up measurements in the UDCA and placebo groups respectively. Data on presenteeism were missing for 47 (3.8 per cent) and 35 (2.7 per cent) follow-up measurements in the UDCA and placebo groups respectively. Missing data on productivity loss were mean imputed by study group and follow-up point.

*Table 3* shows the corresponding healthcare costs as well as non-reimbursable out-of-pocket expenses for patients and productivity losses for employers. The extra costs of preoperative gallbladder ultrasonography and prescription of UDCA (€561) were associated with cost savings for laparoscopic cholecystectomy (–€181, 95 per cent Bca c.i. –€290 to –79) and for emergency department visits (–€72, –119 to –25). The mean costs for formal home care did not differ significantly, but were higher in the placebo group. The mean out-of-pocket expenses for patients were limited, with no difference between study groups. Costs of absenteeism were lower in the UDCA group and costs of presenteeism were lower in the placebo group; the net difference in costs of productivity loss was non-significantly in favour of the UDCA group (–€1046, –3047 to 884).

**Table 3:** Mean costs per patient by study group during patient selection and after discharge from index admission following Roux-en-Y gastric bypass

	Mean cost per patient (€)		
	UDCA group (*n* = 348)	Placebo group (*n* = 357)	Difference UDCA – placebo
**Hospital care**	3135 (2633, 3750)	2884 (2429, 3421)	251 (−506, 1029)
Patient selection and intervention	** **	** **	** **
Preoperative gallbladder ultrasonography	96	0	96
UDCA for 182 days (high price)*	465	0	465
Laparoscopic cholecystectomy	94 (51, 137)	275 (192, 359)	−181 (−290, −79)§
Diagnostic and therapeutic procedures†	1150 (887, 1487)	940 (776, 1118)	210 (−115, 584)
Outpatient specialist consultations	497 (461, 537)	507 (469, 548)	−10 (−64, 45)
Emergency department	129 (103, 155)	200 (165, 239)	−72 (−119, −25)¶
Inpatient stay‡	704 (496, 962)	962 (690, 1313)	−258 (−705, 157)
**Out-of-hospital care**	363 (250, 506)	971 (394, 1761)	−607 (−1572, 43)
Consultations	165 (126, 210)	168 (127, 221)	−3 (−72, 65)
Other institutional care	12 (1, 35)	31 (3, 78)	−19 (−91, 31)
Formal home care	178 (87, 299)	767 (237, 1516)	−588 (−1547, 21)
Use of wound or stoma materials	8 (3, 15)	5 (2, 7)	3 (−3, 11)
**Out-of-pocket expenses**	102 (71, 137)	92 (64, 125)	10 (−39, 58)
**Productivity loss**	6674 (5405, 8010)	7720 (6362, 9273)	−1046 (−3047, 884)
Absenteeism	4744 (3782, 5763)	6028 (4902, 7270)	−1284 (−2899, 244)
Presenteeism	1930 (1446, 2453)	1692 (1282, 2181)	238 (−438, 912)
**Total costs, health care perspective**	3499 (2964, 4150)	3855 (3044, 4822)	−356 (−1573, 761)
**Total costs, societal perspective**	10 274 (8848, 11 772)	11 666 (9935, 13 577)	−1392 (−3807, 917)

Values in parentheses are 95 per cent bias-corrected and accelerated confidence intervals (Bca c.i.).

* The lower price for 182 days of ursodeoxycholic acid (UDCA) prophylaxis was €443. Total and subtotal results (and 95 per cent Bca c.i.) shifted €21 in favour of UDCA prophylaxis.

†Costs of diagnostic and therapeutic procedures were calculated after imputation of group means for 49 patients in the UDCA group and 57 in the placebo group.

‡Included the general ward, ICU, coronary care unit, and day nursing. Costs were weighted by their respective unit costs.

§*P* = 0.001.

¶*P* = 0.002 (independent-samples *t* test).

The mean total costs from a societal perspective were €10 274 (8848 to 11 772) in the UDCA group and €11 666 (9935 to 13 577) in the placebo group. The mean difference of –€1392 (–3807 to 917) in favour of the UDCA group was non-significant. One-quarter of this mean difference was related to the use of healthcare resources. About one-third of the societal costs in both groups were related to the use of healthcare resources.

Patients who developed symptomatic gallstone disease during follow-up generated more costs than those who did not. More than half of their societal costs was generated by the use of healthcare resources. Approximately 87 per cent of the societal costs was attributable to the difference in costs of healthcare (*Table S2*).

### Incremental cost-effectiveness

**Fig. 1:**
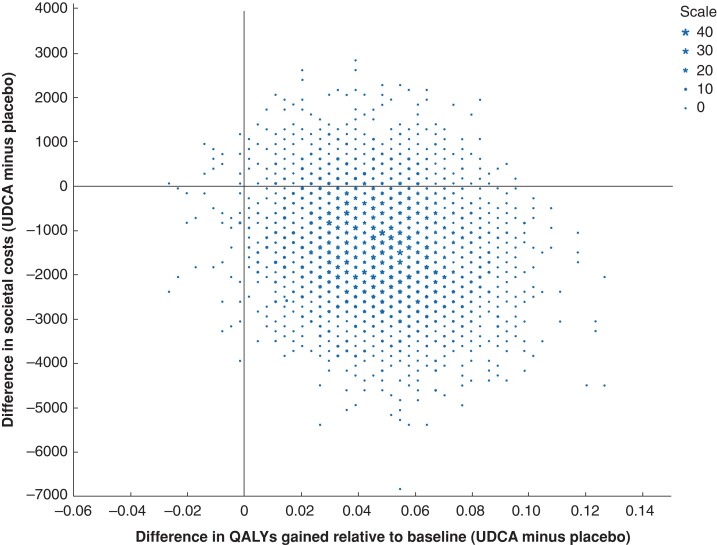
Cost-effectiveness plane showing differences between ursodeoxycholic acid prophylaxis and placebo in societal costs and quality-adjusted life-years gained during 2 years after hospital discharge following Roux-en-Y gastric bypass surgery

UDCA, ursodeoxycholic acid; QALY, quality-adjusted life-year.

**Fig. 2:**
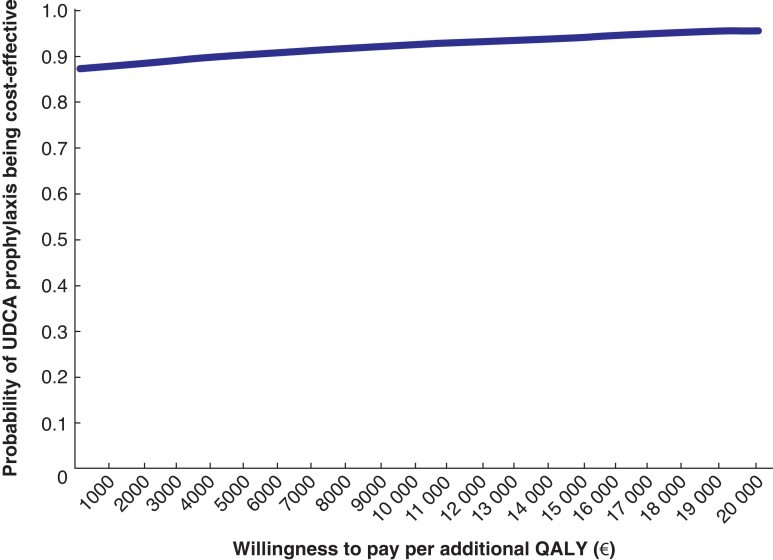
Cost-effectiveness acceptability curve showing the probability of ursodeoxycholic acid being cost-effective at various levels of willingness to pay per additional quality-adjusted life-year up to €20 000

UDCA, ursodeoxycholic acid; QALY, quality-adjusted life-year.

*Figure 1* shows the differences between UDCA prophylaxis and placebo in societal costs and in QALYs gained from baseline after 5000 bootstraps. Considering the point-estimated savings of –€1392 and surplus of 0.047 in QALYs gained from baseline in the UDCA group, most bootstrap results (86.5 per cent) ended up in the lower right quadrant of costs savings and more improved health. Another 12.5 per cent ended up in the upper right quadrant, associated with more improved health at higher costs. The corresponding cost-effectiveness acceptability curve, showing the probability of UDCA being cost-effective at various levels of willingness to pay per additional QALY up to €20 000, is presented in *Fig. 2*. At levels of willingness to pay per extra QALY of €0, €10 000, and €20 000, the probabilities were 0.872, 0.926, and 0.956 respectively. *Figure S1* shows the differences between UDCA prophylaxis and placebo in societal costs and proportion of patients free from symptomatic gallstone disease after 5000 bootstraps, and *Figure S2* the corresponding cost-effectiveness acceptability curve for UDCA prophylaxis at various levels of willingness to pay per patient free from symptomatic gallstone disease. The latter showed that the probability of UDCA being cost-effective was 0.872, 0.912, and 0.942 at €0, €5000, and €10 000 respectively. At a willingness to pay per extra patient free from symptoms of €3000—approximately the unit cost of laparoscopic cholecystectomy—the probability of UDCA prophylaxis being cost-effective was 0.898.

### Discounting

Taking account of the discount rate of 1.5 per cent for QALYs gained during the second year of follow-up, it was noted that patients on UDCA prophylaxis and placebo gained 0.119 (95 per cent Bca c.i. 0.092 to 0.146) and 0.072 (0.041 to 0.102) QALYs over 2 years respectively, with a difference of 0.047 (0.007 to 0.087). The mean total societal costs discounted at a 4 per cent rate were €10 064 (8674 to 11 531) in the UDCA group and €11 430 (9729 to 13 291) in the placebo group, with a mean difference of –€1366 (–3719 to 888). At a 3.5 per cent discount rate, these costs were €10 090 (8695 to 11 559) in the UDCA group and €11 459 (9755 to 13 327) in the placebo group, with a mean difference of –€1369 (–3731 to 893). The discounted differences in mean total costs from a healthcare perspective between the UDCA and placebo groups were –€338 (–1516 to 744) and –€340 (–1525 to 747) at rates of 4 and 3.5 per cent respectively.

## Discussion

This economic evaluation alongside a multicentre RCT showed that UDCA prophylaxis for the prevention of symptomatic gallstone disease after RYGB in patients without gallstones before surgery was cost-effective. UDCA prophylaxis resulted in a significantly higher proportion of patients remaining free from symptomatic gallstone disease and a surplus of nearly 0.05 in QALYs gained from baseline. The mean cost savings of –€1392 was not statistically significant, but was indicative of a sufficient return on investment to compensate for the initially higher upfront costs of patient selection and prophylactic treatment. Hence, from a health economic point of view, UDCA prophylaxis is an efficient drug treatment strategy in at least 87 of every 100 patients without gallstones scheduled for RYGB.

Following a normative approach, the price for the full course of UDCA prophylaxis (182 days) was included despite premature discontinuation of trial medication by some patients. If the exact number of doses of UDCA at patient level had been accounted for, the results would have shifted modestly further in favour of UDCA prophylaxis. Therefore, the reported cost-effectiveness results are slightly conservative.

Patients receiving UDCA prophylaxis underwent cholecystectomy less frequently, had fewer diagnostic and therapeutic procedures, and visited the emergency room less frequently during the 24 months of follow-up. The extra costs of preoperative gallbladder ultrasonography and of prescription of UDCA were largely compensated for by cost savings made from performing fewer cholecystectomies, fewer emergency department visits, and non-significant costs savings of inpatient hospital stay. The *Table S2* in the *supplementary material* provides support for this interpretation by clearly suggesting, based on non-overlapping confidence intervals, that patients who developed symptomatic gallstone disease generated higher costs for these healthcare resources. A higher proportion of these patients was observed in the placebo group. Non-significant savings related to productivity loss further contributed to the efficiency of UDCA prophylaxis.

More counterintuitive findings were the non-significantly higher costs of diagnostic and therapeutic procedures, and the non-significantly lower costs of formal home care, in the UDCA group despite a lower proportion of patients with symptomatic gallstone disease, whereas data in the *Table S2* in the *supplementary material* suggest that these patients had higher costs of diagnostic procedures and lower costs of formal home care. Here, the integral approach of including the use of healthcare resources starting the day after discharge from hospital after bariatric surgery, when resources are still being used as a consequence of the aftermath of such surgery, irrespective of the development and presence of symptomatic gallstones, or as a consequence of co-morbid conditions before and during follow-up (such as having a foot ulcer and subsequent amputations), may clarify these findings. An integral approach, however, is still preferred because QALYs as an outcome measure also reflect the impact of all healthcare consumed.

The time horizon of this economic evaluation was 2 years after the index operation, whereas most economic evaluation guidelines recommend a lifetime horizon for the base-case analysis. However, gallstones tend to develop during the period with the most rapid weight loss, usually the first 6 months after bariatric surgery. Moreover, newly formed gallstones typically become symptomatic in the first 6–18 months after formation^7,10,20^. Therefore, the impact of UDCA prophylaxis in the longer term seems limited. However, underestimation of costs in the placebo group in the longer term is conceivable because of the higher proportion of patients who developed (asymptomatic) gallstones, and, by definition, were more at risk of progressing to symptomatic disease during their lifetime than patients who did not develop gallstones. Of note, the limited time horizon of 2 years meant that the discounting scenarios were of limited impact.

The cost-effectiveness of UDCA prophylaxis for the prevention of gallstones in patients with morbid obesity undergoing rapid weight loss induced by gastric bypass surgery or a very low-calorie diet was investigated previously^21^. In that study, a medical decision analysis model was developed based on data from two landmark clinical trials and on a literature review. The study also showed that UDCA prophylaxis resulted in cost savings. As both clinical trials used gallstone formation as the clinical endpoint of interest, the study had to use estimates of the percentage of patients with symptomatic gallstone disease, and the percentage of patients who would undergo cholecystectomy. Although reliable estimates were used, these comprise a limitation compared with the present study. Furthermore, a time horizon of 1 year was used to simplify the analysis, and a payers’ perspective was chosen. The unit costs used were remarkably higher, which underlines that care should be taken when extrapolating the present results to other countries, because of differences in healthcare systems and unit costs.

Several challenges and limitations of the present economic evaluation should be mentioned. First, the hospital care volume data for patients who underwent the index surgery in one of the bankrupt hospitals needed imputation (73 patients, 10.4 per cent; UDCA group 40, 11.2 per cent; placebo group 33, 9.2 per cent), as well as the data for patients who did not continue regular care in the new hospitals that took over the bariatric surgery departments from the two bankrupt hospitals. Imputation at the subaggregate level of hospital resource use (see methods section) was considered most appropriate. Second, the present hospital volume data were mostly extracted from the hospital information systems of the participating hospitals. Cholecystectomies performed elsewhere were noted on case report forms, and self-reported questionnaires were used to gather data on inpatient hospital stays and emergency departments elsewhere, in addition to gathering data on out-of-hospital care. However, data on diagnostic and therapeutic procedures (other than cholecystectomy) and on outpatient clinic consultations from non-research locations, if any, were missed. In addition, no data were collected on hospital-prescribed medication and personal travel expenses. Ignoring these cost components would probably have affected the results in this analysis to some limited, but actually unknown, extent; if anything, it may have restricted the level of cost-effectiveness of UDCA prophylaxis. Third, a correction had to be made for the baseline difference in health utility. The difference in health utility was also observed in the entire study population of the UPGRADE trial^10^, and could not be explained by observed baseline characteristics. Hence, the baseline difference may have resulted by chance or may have been associated with differences in unobserved baseline characteristics. Without correction for the baseline difference in health utility, no difference in QALYs over time was observed. Yet, because UDCA prophylaxis closed the existing gap at baseline (and regression to the mean as a phenomenon is not to be expected in the absence of extreme scores at group level), the authors considered that correcting for the baseline difference better reflected the benefit of UDCA prophylaxis.

## Supplementary Material

znac273_Supplementary_Data

## References

[1] Lammert F, Gurusamy K, Ko CW, Miquel JF, Mendez-Sanchez N, Portincasa P et al Gallstones. Nat Rev Dis Primers 2016;2:16024 27121416 10.1038/nrdp.2016.24

[2] Adams LB, Chang C, Pope J, Kim Y, Liu P, Yates A. Randomized, prospective comparison of ursodeoxycholic acid for the prevention of gallstones after sleeve gastrectomy. Obes Surg 2016;26:990–994 26342481 10.1007/s11695-015-1858-5

[3] Nabil TM, Khalil AH, Gamal K. Effect of oral ursodeoxycholic acid on cholelithiasis following laparoscopic sleeve gastrectomy for morbid obesity. Surg Obes Relat Dis 2019;15:827–831 31113752 10.1016/j.soard.2019.03.028

[4] Sakran N, Dar R, Assalia A, Neeman Z, Farraj M, Sherf-Dagan S et al The use of Ursolit for gallstone prophylaxis following bariatric surgery: a randomized-controlled trial. Updates Surg 2020;72:1125–1133 32666477 10.1007/s13304-020-00850-2

[5] Altieri MS, Yang J, Nie L, Docimo S, Talamini M, Pryor AD. Incidence of cholecystectomy after bariatric surgery. Surg Obes Relat Dis 2018;14:992–996 29724681 10.1016/j.soard.2018.03.028

[6] Coupaye M, Calabrese D, Sami O, Msika S, Ledoux S. Evaluation of incidence of cholelithiasis after bariatric surgery in subjects treated or not treated with ursodeoxycholic acid. Surg Obes Relat Dis 2017;13:681–685 28089591 10.1016/j.soard.2016.11.022

[7] Portenier DD, Grant JP, Blackwood HS, Pryor A, McMahon RL, DeMaria E. Expectant management of the asymptomatic gallbladder at Roux-en-Y gastric bypass. Surg Obes Relat Dis 2007;3:476–479 17442625 10.1016/j.soard.2007.02.006

[8] Magouliotis DE, Tasiopoulou VS, Svokos AA, Svokos KA, Chatedaki C, Sioka E et al Ursodeoxycholic acid in the prevention of gallstone formation after bariatric surgery: an updated systematic review and meta-analysis. Obes Surg 2017;27:3021–3030 28889240 10.1007/s11695-017-2924-y

[9] Stokes CS, Gluud LL, Casper M, Lammert F. Ursodeoxycholic acid and diets higher in fat prevent gallbladder stones during weight loss: a meta-analysis of randomized controlled trials. Clin Gastroenterol Hepatol 2014;12:1090–1100.e2 24321208 10.1016/j.cgh.2013.11.031

[10] Haal S, Guman MSS, Boerlage TCC, Acherman YIZ, de Brauw LM, Bruin S et al Ursodeoxycholic acid for the prevention of symptomatic gallstone disease after bariatric surgery (UPGRADE): a multicentre, double-blind, randomised, placebo-controlled superiority trial. Lancet Gastroenterol Hepatol 2021;6:993–1001 34715031 10.1016/S2468-1253(21)00301-0

[11] Boerlage TCC, Haal S, Maurits de Brauw L, Acherman YIZ, Bruin S, van de Laar A et al Ursodeoxycholic acid for the prevention of symptomatic gallstone disease after bariatric surgery: study protocol for a randomized controlled trial (UPGRADE trial). BMC Gastroenterol 2017;17:164 29262795 10.1186/s12876-017-0674-x PMC5738131

[12] Haal S, Guman MSS, de Brauw LM, van Veen RN, Schouten R, Fockens P et al Ursodeoxycholic acid for the prevention of symptomatic gallstone disease after bariatric surgery: statistical analysis plan for a randomised controlled trial (UPGRADE trial). Trials 2020;21:676 32703246 10.1186/s13063-020-04605-7 PMC7376318

[13] Welbourn R, Pournaras DJ. Bariatric surgery and prophylaxis against symptomatic gallstone disease. Lancet Gastroenterol Hepatol 2021;6:972–973 34715030 10.1016/S2468-1253(21)00383-6

[14] Husereau D, Drummond M, Petrou S, Carswell C, Moher D, Greenberg D et al Consolidated Health Economic Evaluation Reporting Standards (CHEERS) statement. BMJ 2013;346:f1049 23529982 10.1136/bmj.f1049

[15] Institute for Medical Technology Assessment . https://www.imta.nl/questionnaires/ (accessed 1 November 2021)

[16] Hakkaart-van Roijen L, Van der Linden N, Bouwmans CAM, Kanters TA, Tan SS. *Costing Manual: Methodology of Costing Research and Reference Prices for Economic Evaluations In Healthcare*. 2015 10.1371/journal.pone.0187477 PMC5679627 29121647

[17] Centraal Bureau voor Statistiek . https://opendata.cbs.nl/statline/#/CBS/nl/dataset/83131ned/table?fromstatweb (accessed 1 November 2021)

[18] Versteegh M, Vermeulen K, Evers S, Wit G, Prenger R, Stolk E. Dutch tariff for the five-level version of EQ-5D. Value Health 2016;19:343–352 27325326 10.1016/j.jval.2016.01.003

[19] Barber JA, Thompson SG. Analysis of cost data in randomized trials: an application of the non-parametric bootstrap. Stat Med 2000;19:3219–3236 11113956 10.1002/1097-0258(20001215)19:23<3219::aid-sim623>3.0.co;2-p

[20] Haal S, Rondagh D, Hutten BA, Acherman YIZ, van de Laar AWJM, Huijgen R et al Risk factors for cholecystectomy after laparoscopic Roux-en-Y gastric bypass. Obes Surg 2020;30:507–514 31745862 10.1007/s11695-019-04166-y

[21] Shoheiber O, Biskupiak JE, Nash DB. Estimation of the cost savings resulting from the use of ursodiol for the prevention of gallstones in obese patients undergoing rapid weight reduction. Int J Obes Relat Metab Disord 1997;21:1038–1045 9368828 10.1038/sj.ijo.0800513

