# Supplementary material for: Cost-effectiveness of ursodeoxycholic acid in preventing new-onset symptomatic gallstone disease after Roux-en-Y gastric bypass surgery
Source: Br J Surg. 2022 Aug 18;109(11):1116–23. doi: 10.1093/bjs/znac273 (PMC10364680; doi:10.1093/bjs/znac273)
Supplement: znac273_Supplementary_Data [file znac273_supplementary_data.docx]

| **Table S1. Dutch unit costs (€) for resources used** | | | |
| --- | --- | --- | --- |
| **Resource** | **Unit** | **Unit costs in 2019 euros*** | **Source** |
| Ultrasonography of abdomen† | Procedure | 96.42 | Price of mutual services |
| Ursodeoxycholic acid‡ | Full course , high  Full course, low | 464.59  443.45 | www.medicijnkosten.nl  www.medicijnkosten.nl |
| Laparoscopic cholecystectomy | Procedure | 2977.62 | Hospital ledger |
| Other diagnostic and therapeutic procedures | Procedure | multiple§ | Price for mutual services; hospital ledger |
| Outpatient hospital consultation | Visit  Teleconsult | 97.19  63.57 | Dutch manual for costing  Price of mutual services |
| Emergency department | Visit | 276.61 | Dutch manual for costing |
| Inpatient hospital stay  Ward  High care  Intensive care  Day care | Day  Day  Day  Day | 508.37  1266.65  2346.83  294.77 | Dutch manual for costing  Dutch manual for costing  Price of mutual services  Dutch manual for costing |
| Consultations  General practitioner  Company physician  Psychologist | Visit  Visit  Visit | 35.23  46.97  68.33 | DCM-2015  Dutch manual for costing / expert  Dutch manual for costing |
| Other institution care  Rehabilitation center  Nursing home | Day (adults)  Day | 491.10  179.36 | Dutch manual for costing  Dutch manual for costing |
| Formal home care  Household  Personal care  Nursing at home | Hour  Hour  Hour | 24.56  53.38  77.94 | Dutch manual for costing  Dutch manual for costing  Dutch manual for costing |
| Materials  Wound care  Stoma | Day  Day | 5.34  16.01 | Literature** |
| Out-of-pocket expenses  Over-the-counter medication  Special food / diet  Private home help | Mean monthly costs  Mean monthly costs  Mean monthly costs | as reported  as reported  as reported | Patient  Patient  Patient |
| Productivity loss | Hour | 37.10 | Dutch manual for costing |
| *After price-indexing based on yearly general consumer price indices for the Netherlands. †Although the unit costs of abdominal ultrasonography may exceed the unit costs of an ultrasonography of the gallbladder, one should be aware than the ultrasonography will approximately identify 20% of screened patients who are not eligible for UDCA prophylaxis. Patients scheduled for sleeve gastrectomy should not receive a preoperative gallbladder ultrasonography. ‡Including €14 for dispensing the prescriptions. §For unit costs of diagnostic and therapeutic procedures, please contact the corresponding author. **Ubbink DT, Vermeulen H, Van Hattem J. Comparing of homecare costs of local wound care in surgical patients randomized between occlusive and gauze dressings. J Clin Nursing 2008;17:593-601. | | | |

| **Table S2. Mean costs per patient after discharge from index admission following Roux-en-Y gastric bypass surgery and by study outcome** | | |
| --- | --- | --- |
|  | **Developed symptomatic gallstone disease**  **(n=45)** | **Free of symptomatic gallstone disease**  **(n=660)** |
|  | **Mean costs in €**  (95% Bca CI) | **Mean costs in €**  (95% Bca CI) |
| **Hospital care** | **8673 (7305, 10112)** | **2622 (2291, 3013)** |
| Patient selection & intervention* |  |  |
| Preoperative gallbladder ultrasonography | 26 (15, 36) | 49 (45, 53) |
| UDCA for 182 days | 124 (83, 176) | 237 (218, 255) |
| Laparoscopic cholecystectomy | 2779 (2581, 2911) | 9 (5, 14) |
| Diagnostic and therapeutic procedures | 1842 (1246, 2549) | 989 (834, 1172) |
| Outpatient specialist consultations | 729 (613, 851) | 487 (460, 515) |
| Emergency department | 548 (408, 701) | 139 (117, 161) |
| Inpatient stay† | 2625 (1907, 3379) | 713 (533, 932) |
| **Out-of-hospital care** | **550 (301, 859)** | **679 (368, 1081)** |
| Consultations | 374 (209, 578) | 153 (123, 186) |
| Other institutional care | 6 (1, 13) | 23 (3, 49) |
| Formal home care | 154 (20, 377) | 498 (208, 884) |
| Use of wound or stoma materials | 16 (6, 29) | 6 (2, 10) |
| **Out-of-pocket expenses** | **113 (43, 197)** | **96 (73, 120)** |
| **Productivity loss** | **8008 (4994, 11215)** | **7149 (6159, 8199)** |
| Absenteeism | 6848 (4180, 9615) | 5295 (4497, 6155) |
| Presenteeism | 1160 (442, 2082) | 1854 (1517, 2230) |
| **Total costs, health care perspective** | **9222 (7762, 10721)** | **3301 (2792, 3903)** |
| **Total costs, societal perspective** | **17343 (13869, 20903)** | **10545 (9345, 11822)** |
| UDCA=ursodeoxycholic acid. *Point-estimated mean costs of ultrasonography and UDCA are lower in the group of patients who developed symptomatic gallstone disease because of overrepresentation of the placebo group as a consequence of the prophylactic impact of UDCA. †Inpatient stay included the general ward, intensive care unit, coronary care unit, and day nursing. Costs were weight by their respective unit costs. | | |

**Figure S1. Cost-effectiveness plane showing differences between UDCA prophylaxis and placebo in societal costs and proportion of patients free of symptomatic gallstone disease during two years after hospital discharge following Roux-en-Y gastric bypass surgery**

**
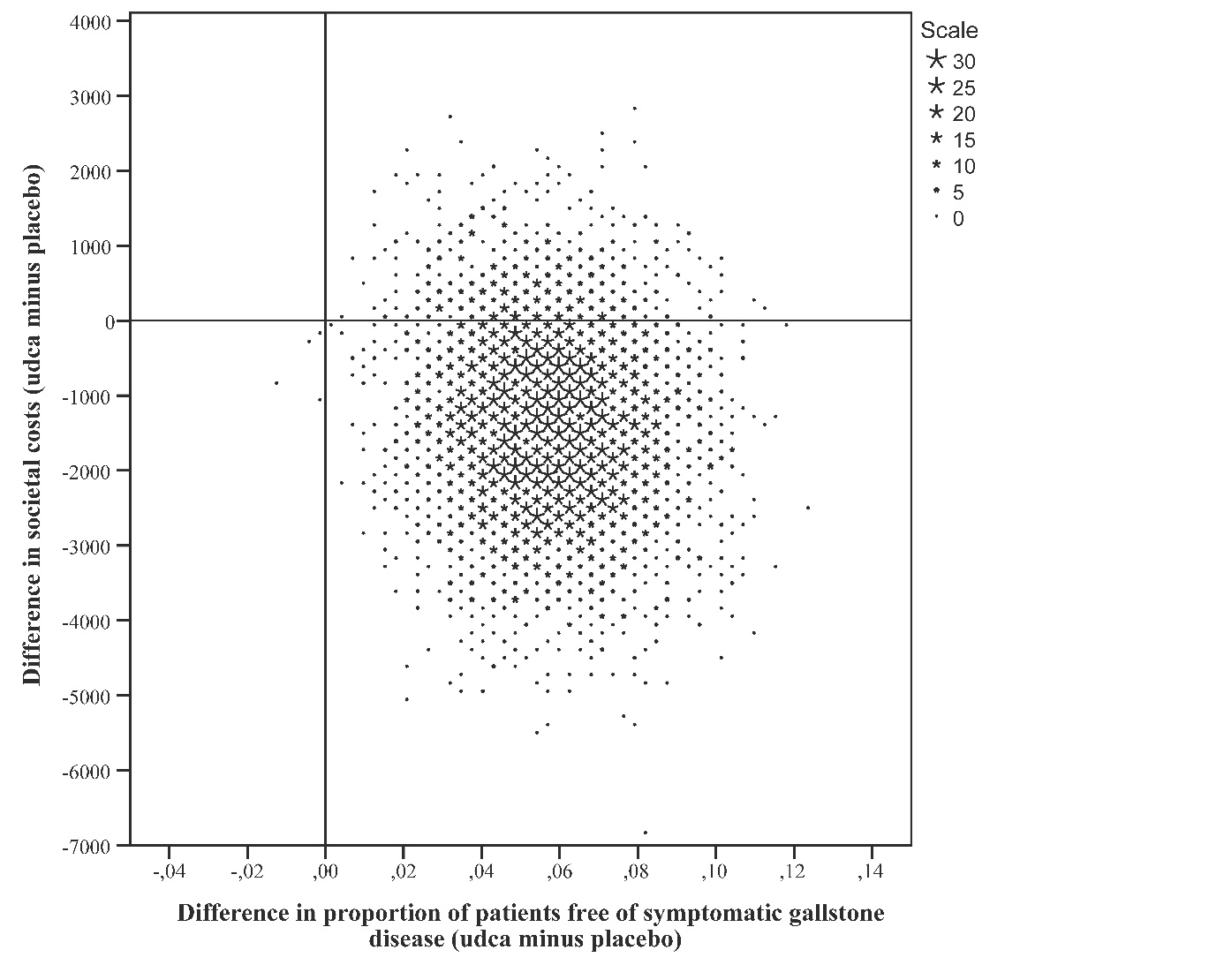
**

UDCA=ursodeoxycholic acid.

**Figure S2. Cost-effectiveness acceptability curve showing the probability of ursodeoxycholic acid being cost-effective at various levels of willingness to pay per patient free of symptomatic gallstone disease up to €10,000**

UDCA=ursodeoxycholic acid.
